# Second Palearctic Record of the Genus *Stereoglyphus* Berlese (Acari: Acaridae) with Morpho-Molecular Description of a New Species from Zagros Mountains, Iran †

**DOI:** 10.3390/insects17030237

**Published:** 2026-02-25

**Authors:** Mojgan Sadat-Shojaei, Miroslawa Dabert, Mohammad Ali Akrami, Saber Sadeghi, Jacek Dabert

**Affiliations:** 1 Department of Biology, College of Science, Shiraz University, Shiraz P.O. Box 7145685464, Iran; m.sadatshojaei@shirazu.ac.ir; 2 Molecular Biology Techniques Laboratory, Faculty of Biology, Adam Mickiewicz University, Uniwersytetu Poznanskiego 6, 61-614 Poznan, Poland; miroslawa.dabert@amu.edu.pl; 3 Department of Plant Protection, School of Agriculture, Shiraz University, Shiraz P.O. Box 7144113131, Iran; 4 Department of Animal Morphology, Faculty of Biology, Adam Mickiewicz University, Uniwersytetu Poznanskiego 6, 61-614 Poznan, Poland

**Keywords:** Astigmata, ontogeny, subterranean, troglobiont, burrowing adaptation, cave, COI, SEM

## Abstract

In this study, the astigmatid mite genus *Troglocoptes* Fain, 1966 is proposed as a junior synonym of *Stereoglyphus* Berlese, 1923. As a part of the project concerning identification of cave-dwelling mites in the Zagros Mountains, all ontogenetic instars of *Stereoglyphus iranensis* sp. nov. (Sarcoptiformes: Acaridae) are described from Doroodzan Cave, Fars Province, Iran. This is the second record of the genus in caves in the Palearctic region and the fifth described species worldwide. The morphological description is supplemented with DNA barcode data based on the mitochondrial cytochrome *c* oxidase subunit I (COI) gene, representing the first molecular data for this genus. Additionally, the first Asian record of *Stereoglyphus longibursatus* (Fain et Mahunka, 1990) is reported from Sahlak Cave, Fars Province, Iran. An identification key to the known species of the genus is provided. The troglobitic status of the new species is discussed, and the modifications of the anterior legs and tarsal setae, along with the partial reduction of idiosomal setation, are interpreted as adaptations to burrowing in bat guano.

## Simple Summary

Until now, the acarofauna of caves in the Zagros Mountains, Iran, has been poorly recognized, although cavernicolous astigmatid mites are cosmopolitan and are typically associated with dark and humid subterranean environments. This study presents part of the results of the first comprehensive survey conducted in this region. Comparative analysis of the sampled mites with literature data and type collections has led to the conclusion that the genus *Troglocoptes* Fain, 1966 should be assigned as a junior synonym of *Stereoglyphus* Berlese, 1923. We found two species of this genus, including one species new to science; this represents the second record of the genus *Stereoglyphus* in the Palearctic. The description of the new species is based on morphological examination of all life stages and is supplemented by DNA barcoding data. The identification key to the known species of the genus is provided.

## 1. Introduction

Mites of the family Acaridae (Acariformes: Astigmata) are cosmopolitan and many species feed on decomposing organic matter. They are found in a variety of habitats, including caves [1,2]. One of the several genera of acaridid mites recorded from cave habitats is *Stereoglyphus* Berlese, 1923, originally described from a cave in Calcutta, India [3]. Berlese designated the only included species *S. haemisphaericus* as the type species based solely on the female specimen, as the male was not present in the type series. Much later, Fain described another cave-dwelling genus *Troglocoptes* Fain, 1966, based on material collected from Tordeur Cave, Democratic Republic of Congo [4]. At that time, the genus was monotypic, with *T. luciae* designated as the type species. As in the previous case, the description was based solely on female specimens, as no males were found. In subsequent studies, Fain described two additional species of *Troglocoptes*: *T. subterraneus* Fain, 1976 from Amsterdam Island, French Southern and Antarctic Lands [5], and *T. longibursatus* Fain et Mahunka, 1990 from Kiskunság National Park, Hungary [6]. In these cases, the descriptions were complete and included diagnoses of both males and females. Despite more than 100 years having elapsed since the description of *Stereoglyphus haemisphaericus* and 60 years since the description of *Troglocoptes luciae*, the males of these species remain unknown.

When describing the genus *Troglocoptes*, Fain provided a differential diagnosis with *Schwiebea* Oudemans, 1923, which he considered to be the morphologically closest genus. Furthermore, in a subsequent work, he provisionally assigned another cave-dwelling species, *Schwiebea barbei* Cooreman, 1959, to the genus *Troglocoptes* [7]. It is likely that he did not have access to the type series of *Stereoglyphus*, as Berlese’s brief description, devoid of illustrations, neither allowed for a meaningful comparative analysis nor suggested any similarity between *Stereoglyphus* and *Troglocoptes*.

Several years ago, OConnor, University of Michigan, a distinguished expert on the taxonomy, biology, and ecology of astigmatid mites, examined the type material of *Stereoglyphus haemisphaericus* in the Berlese collection and compared it with his previously undescribed material from the Philippines and North America and literature data. He stated that Fain’s descriptions of the genus *Troglocoptes* were fully consistent with the morphology of *S. haemisphaericus*, and therefore *Troglocoptes* should be regarded as a junior synonym of *Stereoglyphus*. This conclusion was informally expressed for nearly 30 years, including in OConnor’s unpublished teaching materials and on a scientific discussion blog [8]. With the written consent of OConnor [9], we have gained access to this information and formally synonymize the two genera in the present study.

Acarofauna of troglobiont astigmatid mites of Iran is poorly known [10] and this study presents partial results of the project aimed at identifying subterranean Acari of caves located in the Zagros Mountains, Iran. In this survey, two species of cave-dwelling astigmatid mites of the genus *Stereoglyphus* Berlese, 1923 were collected from the hypogean zone of Doroodzan Cave (a species new to science) and of Sahlak Cave (*S. longibursatus*, a new record for Asia). The description of the new species was based on microscopic morphological analysis supported by scanning electron microscopy (SEM) and supplemented with DNA barcode sequences; the COI sequences represent the first molecular data for this genus. The description also includes an analysis of the morphology of juvenile stages, which has not previously been investigated for this genus.

## 2. Materials and Methods

### 2.1. Sampling and Documentation

Sampling was conducted from a mixture of soil and bat guano in the Doroodzan Cave and Sahlak Cave located in the Zagros Mountains, Fars Province, Iran. Most of the caves in this region are limestone. Doroodzan Cave is a natural karstic pit cave that requires vertical caving, located in Marvdasht County, Doroodzan District (30°14′56.695″ N, 52°22′37.275″ E), that is almost 5 m long, 47.7 m deep and 1740 m in height above mean sea level. Sampling was carried out once in this cave on 22 April 2022. Sahlak Cave is a natural horizontal karstic formation measuring nearly 330 m in length, located in Darab County, Chah Kondar Village (28°32′51.20″ N, 55°08′33.80″ E). Sampling was performed once on 4 December 2021. Mixtures of soil and bat guano were sampled in all three zones of both caves: the endogean (entrance zone), which receives the most light and exhibits highly variable temperatures; the parahypogean (twilight zone), with less light and temperature variation; and the hypogean (dark zone), characterized by nearly constant temperatures and maximum humidity. The species studied in this research were both collected from the dark hypogean zones of the caves mentioned. The fauna previously recorded in Sahlak Cave includes bats from the families Rhinolophidae (*Rhinolophus ferrumequinum*, *R. hipposideros*, *R. blasii*), Miniopteridae (*Miniopterus pallidus*) and Vespertilionidae (*Myotis blythii*) [11]. Arthropods such as coleopterans *Gibbium aequinoctiale* (Ptinidae) [12], *Blaps bushirensis* (Tenebrionidae) [10], *Laemostenus* (*Antisphodrus*) *sahlakensis* (Carabidae) [13], orthopteran *Eremogryllodes iranicus* (Myrmecophilidae) [14], isopod *Protracheoniscus flavescens* (Agnaridae) [15] and sarcoptiform mite *Ciprusenia troglobionta* (Canestriniidae) [10] have also been reported from this cave. The fauna of Doroodzan Cave has not yet been inventoried.

In the laboratory, mite specimens were extracted using Berlese–Tullgren funnels. Soiling of the mite bodies, including fungal, soil, and bat guano particles, was removed with fine needles.

Mounted microscopic slides were prepared from all life stages, excluding inert deutonymphs. To prepare the slides, the specimens were cleared in Nesbitt’s fluid for one to two hours (depending on the life stage and the degree of the body sclerotization). Then, the specimens were transferred into a drop of Hoyer’s medium on the slide with a small handmade loop tool. The appropriate position of the specimen’s body was created with a fine needle, and finally, coverslipping was performed. The slides were kept in an oven at 45 °C for two weeks, and then labeled [16].

Several mounted microscopic slides, including all life stages, were photographed using a Nikon Eclipse 80i microscope equipped with a digital camera.

Identification and manual line drawings of the specimens were created using an Olympus BX53 DIC microscope equipped with a camera lucida.

Ten female specimens were used for scanning electron microscope (SEM) photography. For fixation, after removing surface soiling, the specimens were preserved in 70% ethanol for 10 days. After washing the specimens with distilled water, they were preserved in a solution of 70% ethanol with a drop of glycerol for one hour. Then, each specimen was carefully transferred to the conductive double-sided adhesive on the stub using a fine brush. The stub containing the specimens was left overnight at room temperature in a clean, dry box with the lid closed. Finally, the stub was coated with Q 150R-ES (Quorum Technologies, Laughton, England) sputtering coating and photography was performed with the TESCAN-VEGA 3 (TESCAN, Brno, Czech Republic) scanning electron microscope [17].

Adobe Photoshop ver. 24.1.0 (Adobe Inc., San Jose, CA, USA, 2023) was used to edit all images, including the initial drawings and microscopic output photos. All measurements are given in micrometers (μm) and were performed using ImageJ ver. 1.54g (National Institutes of Health, Bethesda, MD, USA, 2023). A map of the known geographical distribution of the genus *Stereoglyphus* was created in QGIS ver. 3.44.3-Solothurn (Free Software Foundation, Inc., Boston, MA, USA, 2025).

The morphological terminology and abbreviations for idiosomal chaetotaxy follow the system proposed by Griffiths et al. [18] and modified by Norton [19], the palpal and leg chaetotaxy follow Grandjean [20] and Griffiths et al. [21], and the genital structures terminology follows that of Klimov et OConnor [22].

Abbreviations of depositories: ZM-CBSU—Zoological Museum, Collection of Biology Department, Shiraz University, Shiraz, Iran; JAZM—Jalal Afshar Zoological Museum, Department of Plant Protection, University of Tehran, Karaj, Iran; AMU—Molecular Biology Techniques Laboratory, Faculty of Biology, Adam Mickiewicz University, Poznan, Poland.

### 2.2. DNA Extraction and PCR Amplification

Eleven female specimens were individually transferred from 96% ethanol into Eppendorf tubes containing 180 μL of ATL lysis buffer (Qiagen, Hilden, Germany) for DNA extraction. Subsequently, 20 μL of 2 mg/mL Proteinase K (Novazym, Poznan, Poland) was added, and the samples were incubated at 56 °C with shaking (200 rpm) for 4 to 7 days, depending on the degree of sclerotization of the mites. Total genomic DNA of the specimens was extracted individually as described by Dabert et al. [23]. After DNA extraction, the mite exoskeletons were transferred into tubes containing 70% ethanol for subsequent microscopic slide preparation. The mounted specimens were retained as voucher material and used for comparative analyses of morphological characters.

For DNA barcoding, the COI gene fragment was amplified by PCR in 10 μL reaction volumes, containing 2 μL of HOT FIREPol Blend Master Mix (Solis BioDyne, Tartu, Estonia), 0.5 μM each primer (Table 1), and 4 μL of DNA template. The thermocycling conditions were as follows: initial denaturation at 95 °C for 12 min, then 40 cycles of denaturation at 95 °C for 15 s, annealing at 50 °C for 1 min, extension at 72 °C for 1 min, and final extension at 72 °C for 5 min.

**Table 1:** Primers used in this study: a and s refer to amplifying and sequencing, respectively.

Primer	Sequence	Product	Used	Source
bcdF05	TTTTCTACHAAYCATAAAGATATTGC	COI	a, s	[23]
bcdR04	TATAAACYTCDGGATGNCCAAAAAA	COI	a, s	[23]

After amplification, samples were diluted 2 times with MQ water and 5 μL were analyzed by electrophoresis on a 1% agarose gel. Samples with visible bands were purified using thermosensitive Exonuclease I and FastAP Alkaline Phosphatase (Thermo Fisher Scientific, Waltham, MA, USA). Amplicon sequencing was carried out using BigDye Terminator v3.1 (Applied Biosystems, Foster City, CA, USA) chemistry on an ABI Prism 3130XL Analyzer (Applied Biosystems). Sequence chromatograms were checked and edited with Chromas ver. 2.6.6 (Technelysium Pty Ltd., South Brisbane, Australia, 2019) and COI contigs were assembled and aligned with GeneDoc ver. 2.7.000 [24].

## 3. Results

### 3.1. Taxonomic Emendations

Superfamily Acaroidea Latreille, 1802.

Family Acaridae Latreille, 1802.

Subfamily Rhizoglyphinae Zakhvatkin, 1941.

Genus *Stereoglyphus* Berlese, 1923.

*Stereoglyphus* Berlese, 1923: 261 [Type species *Stereoglyphus haemisphaericus* Berlese, 1923, by monotypy].

*Troglocoptes* Fain, 1966: 397 [Type species *Troglocoptes luciae* Fain, 1966, by monotypy], syn. nov.

Based on comparison of *Troglocoptes* Fain, 1966 original description [4] with the type material of *Stereoglyphus* Berlese, 1923 made by Barry OConnor [9], and also descriptions of remaining *Troglocoptes* species [5,6] and on our own additional material we concluded that these two genera are congeneric. Therefore, we transfer *Troglocoptes* to the genus *Stereoglyphus* and consider *Troglocoptes* syn. nov. as a junior synonym of this genus.

**Genus diagnosis.** Dorsal idiosoma fully sclerotized, in adult punctate, with large pronotal and hysteronotal shields separated by well-developed sejugal furrow. Setae *ve*, *c*_3_, *f*_2_ absent, remnants of setae *si* and *c*_1_ may be present only in the form of vestigial alveolae; large latero-dorsal slit-shaped cupulae *ia* observed near setae *cp*. Legs short and thick, I-II distinctly more massive than III-IV, with tarsal setae (except filiform *d* and *ra*) shaped as short spines, *e* and *ba* larger than remaining ones.

### 3.2. New Species Description

***Stereoglyphus iranensis* Sadat-Shojaei, Akrami & Dabert J. sp. nov.** urn:lsid:zoobank.org:act:AF3155ED-180D-4D9C-BDFD-ADF519C3F402.


**Material examined.** Male holotype, 29 female paratypes including 10 for SEM, 11 for DNA isolation and 8 for slide mounting, additional material including 6 tritonymphs, 3 protonymphs, 1 larva, Iran, Fars Province., Marvdasht County, Doroodzan District, Doroodzan Cave; 30°14′56.695″ N, 52°22′37.275″ E; mixture of soil and bat guano; 22 April 2022; leg. M. Sharifi.

**Depository.** Male holotype, 6 female paratypes, 4 tritonymphs, 2 protonymphs, 1 larva, microscopic slides, 10 female paratypes, coated stub, 11 female paratypes, voucher microscopic slides, (ZM-CBSU); 2 female paratypes, 2 tritonymphs, 1 protonymph, microscopic slides, (JAZM); 11 female paratypes, DNA isolates, (AMU).

**Etymology.** The species name *iranensis* refers to Iran, a country in Southwest Asia, where the type specimens were collected.

**Figure 1:**
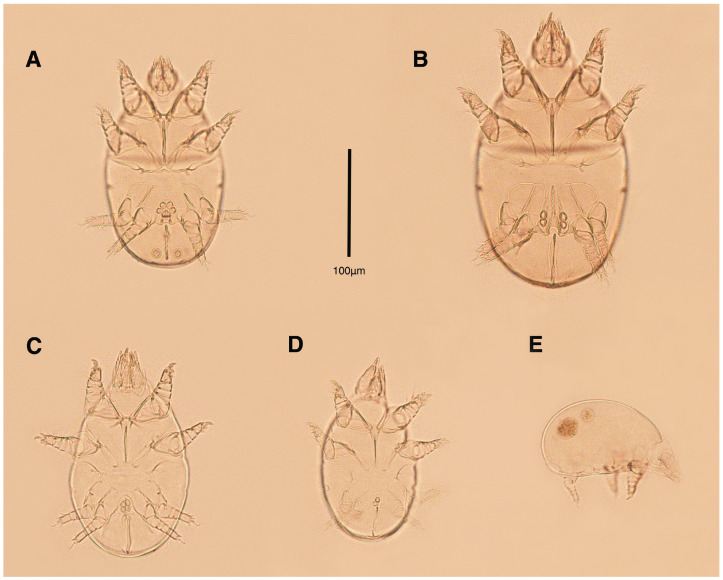
*Stereoglyphus iranensis* sp. nov., optical micrographs of mounted microscopic slides; (**A**) male, holotype; (**B**) female, paratype; (**C**) tritonymph; (**D**) protonymph; (**E**) larva.

Male, holotype.

Gnathosoma. (Figure 3B). Elongated trapezoid; palpal tarsi with dorso-subterminal spiniform setae *cm*, rod-shaped solenidia *ω* with curved head, button-shaped setae *ul*^′^, *ul*^″^; palpal tibiae dorsally with setae *sup*, ventrally with setae *a*; subcapitular setae *h* present; setae *elcp* spiniform and barely visible; chelicerae chelate-dentate with 4–5 teeth on each digit, movable digits with spiniform setae *cha*; setae *sup*, *a*, *h*, all simple filiform; measurements: length of gnathosoma 42, width of gnathosoma 30; palpal length 12; cheliceral length 31; *cha* 2; *elcp* 2; *h* 11, *h*–*h* 9; *sup* 6; *a* 4; *cm* 4; *ω* 4.

**Figure 2:**
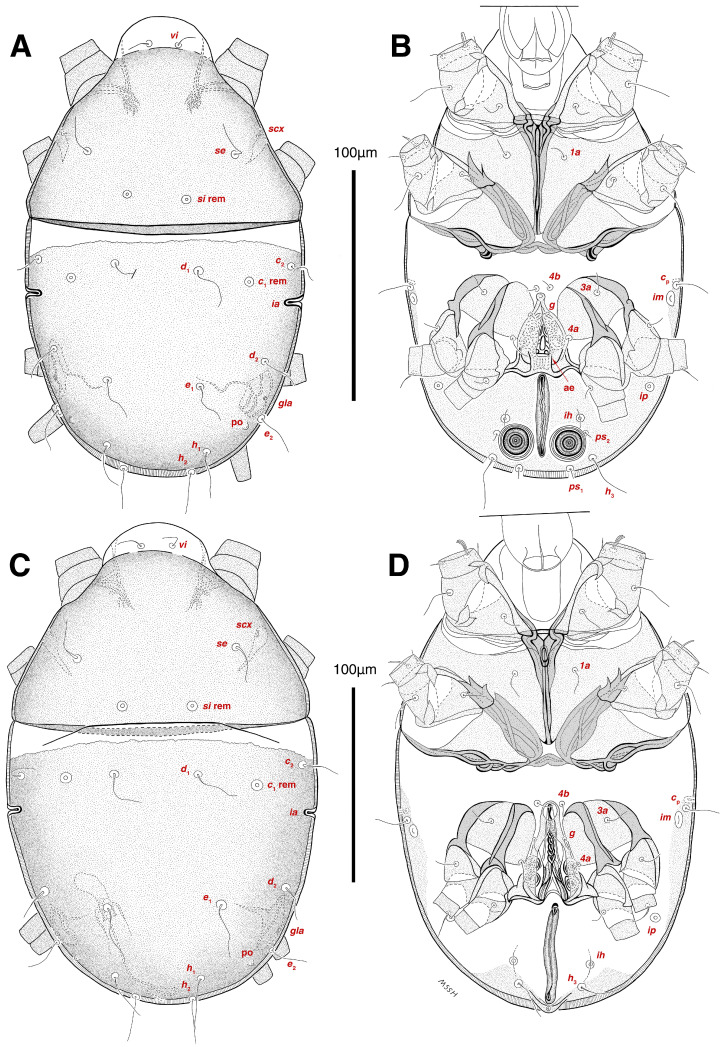
*Stereoglyphus iranensis* sp. nov. Male, holotype, (**A**) dorsal view, (**B**) ventral view; female, paratype, (**C**) dorsal view, (**D**) ventral view. Abbreviations: rem—setal remnant, po—porus, ae—aedeagus. Segments and setae of the legs are not shown in full.

**Figure 3:**
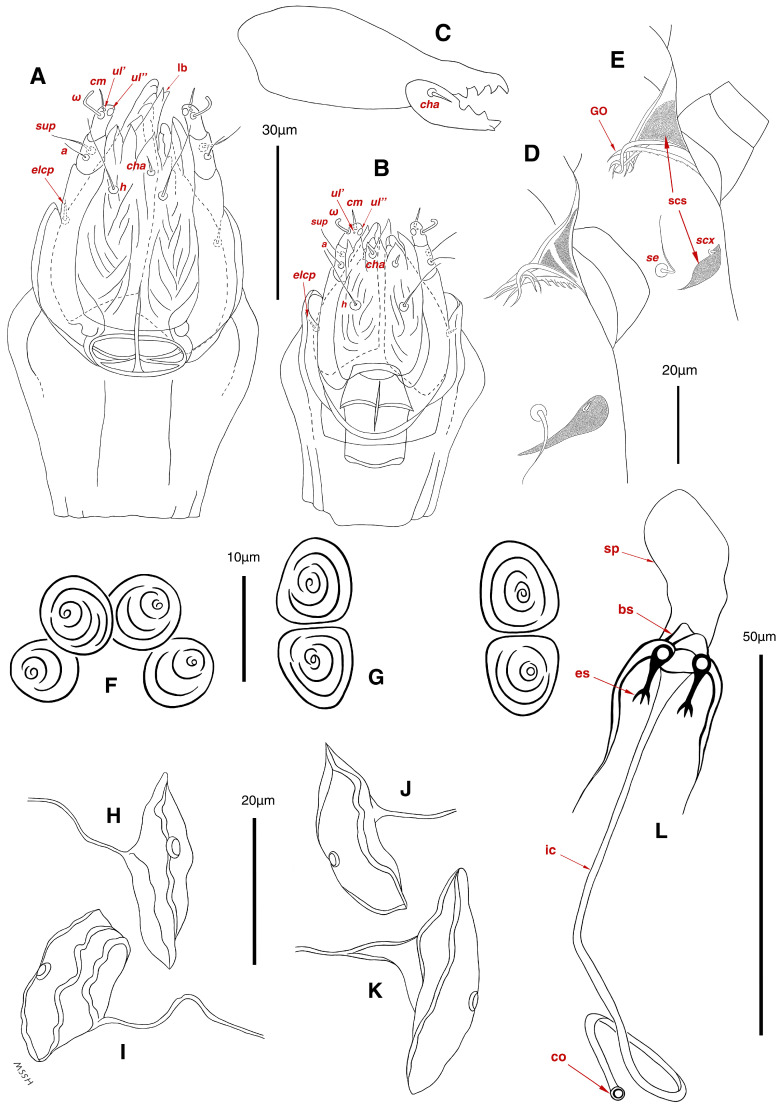
*Stereoglyphus iranensis* sp. nov., details. (**A**) Gnathosoma of female and (**B**) male; (**C**) chelicera of female; (**D**) Grandjean’s organ, supracoxal sclerites and seta *scx* of female and (**E**) male; (**F**) genital papillae of male and (**G**) female; (**H**,**I**) right and left *gla* of male and female (**J**,**K**) female; (**L**) bursa copulatrix. Abbreviations: lb—labrum, GO—Grandjean’s organ, scs—supracoxal sclerite, sp—spermatheca, bs—base of spermatheca, es—efferent spermaduct, ic—inseminatory canal, co—copulatory opening.

Dorsum. (Figure 1A, Figure 2A, Figure 3E,H,I and Figure 5A). Idiosoma dorsally convex and ovoid in outline, completely sclerotized and finely punctate except anterior part of propodosoma and a narrow part around sejugal furrow separating large pronotal and hysteronotal shields (Figure 1A and Figure 2A); Grandjean’s organs well-developed, multi-branched, along with anterior punctate supracoxal sclerites; setae *scx* present, small and conical, on posterior punctate supracoxal sclerites (Figure 2A, Figure 3E and Figure 5A); remnants of missing setae *si* and *c*_1_ well visible as rounded cupuli-form stigmata (Figure 2A); on hysterosoma a pair of cupule *ia* shaped as big slit-like cupulae with distinct margins and placed dorsolaterally, posterior to setae *c*_2_; opisthonothal glands and their openings, *gla*, well-developed, located near posterolateral body margin, between bases of setae *d*_2_ and *e*_2_ (Figure 2A and Figure 3H,I); a pair of unidentified pori observed posterior to setae *e*_2_ near body margin; setae *h*_1_ observed posterior to opisthosoma; setae *h*_2_ located posterior to setae *h*_1_, on posterior margin of opisthosoma; posterior margin of opisthosoma rounded (Figure 2A); all dorsal idiosomal setae simple filiform; measurements: idiosoma length 207, idiosoma width 124; propodosomal length 90; *vi* 9, *vi*–*vi* 13; *se* 18, *se*–*se* 63; *scx* 2, *scx*–*scx* 84; *d*_1_ 22, *d*_1_–*d*_1_ 36; *c*_2_ 12, *c*_2_–*c*_2_ 110; *d*_2_ 17, *d*_2_–*d*_2_ 91; *e*_1_ 19, *e*_1_–*e*_1_ 40; *e*_2_ 19, *e*_2_–*e*_2_ 88; *h*_1_ 20, *h*_1_–*h*_1_ 43; *h*_2_ 18, *h*_2_–*h*_2_ 29; *gla*–*gla* 92; *ia* length 7.

Venter. (Figure 1A, Figure 2B, Figure 3F and Figure 5E,F). Idiosoma nearly flat on ventral surface, not punctate and sclerotized in median part; all coxal apodemes well-developed with fused coxal fields covered by extensive shield; setae *c*_p_ placed near slit-shaped cupulae, *ia*; cupulae *im* located ventrolateral, slightly posterior to setae *c*_p_; cupulae *ip* placed ventrolateral at opisthosoma margin, between legs III and IV (Figure 1A and Figure 2B); genital valves appear as transparent layers covering on genital papillae and aedeagus; genital papillae well-developed, anterior pair adjoining, posterior pair separated by 6, each anterior and corresponding posterior papilla adjoining, diameter of each genital papilla 7; small genital setae, *g*, set anterolateral to genital capsule in middle-distance *4a*–*4b*; cupulae *ih* positioned anterior to setae *ps*_2_, with a vertical suture on them (Figure 2B, Figure 3F and Figure 5E); anal slit long with punctate covering, extends from posterior end of genital area to level of posterior margin of anal suckers; well-developed pair of anal suckers set close to posterior margin of opisthosoma, each with multidentate corollas, 15 in diameter; setae *ps*_1_ and *ps*_2_ positioned at posterior and anterolateral margin of anal suckers, respectively; setae *h*_3_ placed at posterior margin of anal suckers, laterally; opisthoventrum more finely punctate than dorsum; posterior margin of opisthosoma rounded (Figure 2B and Figure 5F); all ventral idiosomal setae simple filiform; measurements: *1a* 7, *1a*–*1a* 25; *3a* 9, *3a*–*3a* 49; *4a* 11, *4a*–*4a* 24; *4b* 4, *4b*–*4b* 6; *c*_p_ 15, *c*_p_–*c*_p_ 115; *g* 3, *g*–*g* 7; *h*_3_ 22, *h*_3_–*h*_3_ 43; *ps*_1_ 4, *ps*_1_–*ps*_1_ 22; *ps*_2_ 3, *ps*_2_–*ps*_2_ 38; *im*–*im* 111; *ip*–*ip* 91; *ih*–*ih* 32; anal slit length 36.

**Figure 4:**
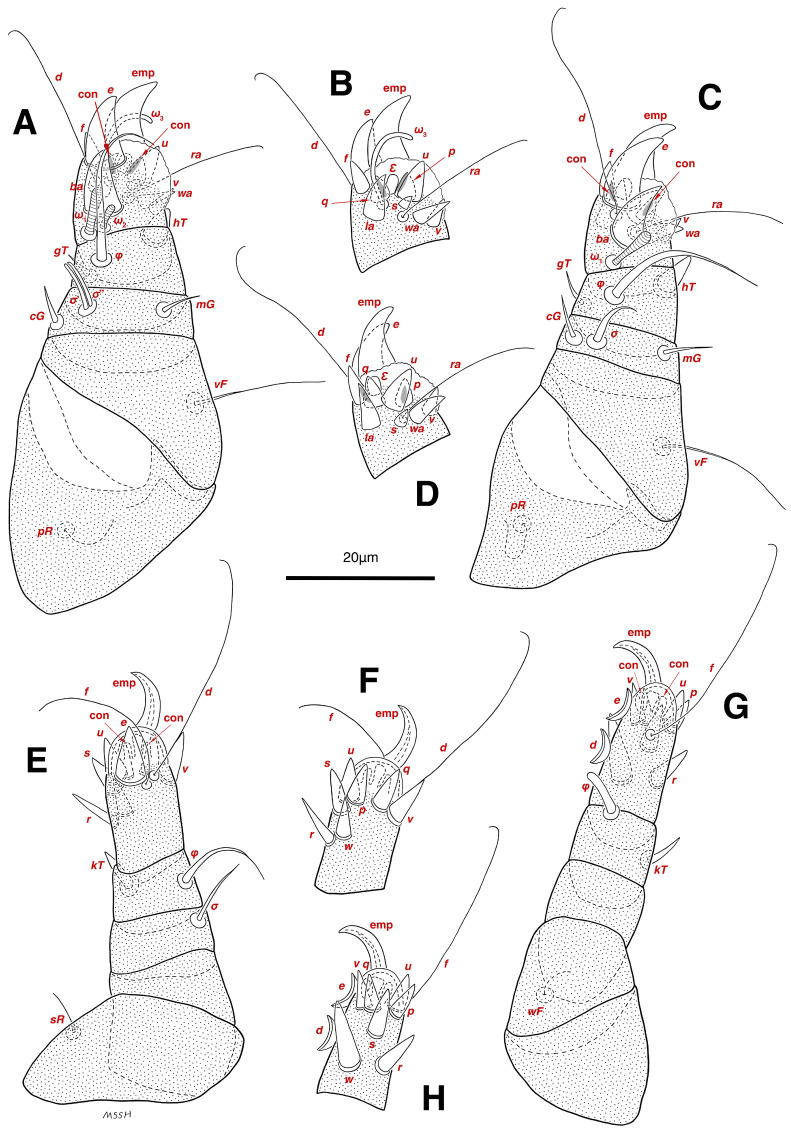
*Stereoglyphus iranensis* sp. nov., legs of male. (**A**) Leg I, dorsal view, (**B**) tarsus I, ventral view; (**C**) leg II, dorsal view, (**D**) tarsus II, ventral view; (**E**) leg III, dorsal view, (**F**) tarsus III, ventral view; (**G**) leg IV, dorsal view, (**H**) tarsus IV, ventral view. Abbreviations: emp—empodial claw, con—condylophore.

**Figure 5:**
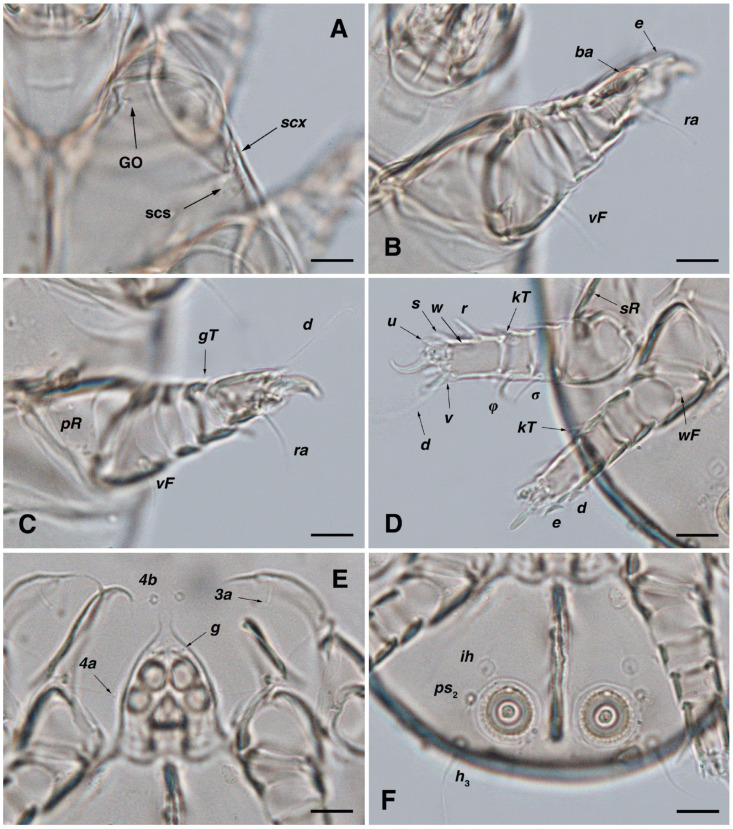
*Stereoglyphus iranensis* sp. nov., optical micrographs of male. (**A**) Grandjean’s organ, seta *scx*, supracoxal sclerite; (**B**) leg I; (**C**) leg II; (**D**) leg III–IV; (**E**) genital area; (**F**) opisthosoma. Abbreviations: GO—Grandjean’s organ, scs—supracoxal sclerite. Scale bar: 10 μm.

Legs. (Figure 4A–H and Figure 5B–D and Table 2). Legs I–II stout, distinctly thicker than legs III–IV; vestigial pulvilli and small condylophores visible in all pretarsi; all legs punctate except paraxial part of trochanters of legs I–II; empodial claws of tarsi I–II stout hooked, setae *d*, *ra* filiform, setae *e*, *ba* stout conical slightly hooked, famuli *Ԑ* with blunt tip positioned subapical, longer solenidia *ω*_1_ and shorter solenidia *ω*_2_ placed basal, curved solenidia *ω*_3_ observed subapical; genual solenidia *σ*′, *σ*″ equal; empodial claws of tarsi III–IV long and hooked, setae *f* and *d* of tarsi III filiform; on tarsi IV setae *f* filiform, setae *e*, *d* sucker-like; chaetotaxy of legs I–IV: trochanters 1-1-1-0, femora 1-1-0-1, genua 2(2*σ*)-2(1*σ*)-(1*σ*)-0, tibiae 2(1*φ*)-2(1*φ*)-1(1*φ*)-1(1*φ*), tarsi 12(3*ω*,1*Ԑ*)-12(1*ω*,1*Ԑ*)-10-10; measurements (including pulvilli, excluding empodial claws): leg I 66, leg II 62, leg III 59, leg IV 61; trochanter I 28, *pR* 9, femur I 13, *vF* 18, genu I 6, *σ*′ 7, *σ*″ 7, *mG* 5, *cG* 5, tibia I 8, *φ* 23, *hT* 5, *gT* 4, tarsus I 12, *ω*_1_ 6, *ω*_2_ 3, *ω*_3_ 13, *Ԑ* 3, *ba* 9, *wa* 5, *la* 6, *v* 4, *d* 24, *ra* 20, *e* 3, *q* 4, *p* 4, *u* 6, *f* 5, *s* 2, condylophore 3, empodial claw 14; trochanter II 24, *pR* 11, femur II 12, *vF* 19, genu II 5, *σ* 8, *mG* 6, *cG* 6, tibia II 8, *φ* 22, *hT* 6, *gT* 5, tarsus II 12, *ω*_1_ 6, *Ԑ* 3, *ba* 9, *wa* 5, *la* 6, *v* 4, *d* 30, *ra* 20, *e* 11, *q* 4, *p* 4, *f* 5, *s* 2, condylophore 2, empodial claw 13; trochanter III 24, *sR* 6, femur III 6, genu III 6, *σ* 9, tibia III 6, *φ* 14, *kT* 6, tarsus III 6, *w* 5, *r* 7, *d* 32, *f* 22, *e* 7, *p* 5, *q* 5, *u* 6, *v* 6, *s* 6, condylophore 2, empodial claw 10; trochanter IV 16, femur IV 13, *wF* 5, genu IV 7, tibia IV 8, *φ* 6, *kT* 5, tarsus IV 18, *w* 9, *r* 6, *d* 5, *f* 33, *e* 5, *p* 5, *q* 5, *u* 5, *v* 5, *s* 6, condylophore 2, empodial claw 10.

Female (*n* = 29), paratypes.

Gnathosoma. (Figure 3A,C, Figure 7A and Figure 11A–C). Elongated trapezoid; part of labrum visible; spiniform setae *cm*, rod-shaped solenidia *ω* with curved head, button-shaped setae *ul*^′^, *ul*^″^ observed on subterminal of palpal tarsi; setae *sup* dorsally and setae *a* ventrally positioned palpal tibiae; subcapitular setae *h* present; spiniform setae *elcp* visible with difficulty; chelicerae chelate-dentate with 4–5 teeth on each digit, movable digits with spiniform setae *cha*; all setae *sup*, *a*, *h* simple filiform; measurements: length of gnathosoma 51–55, width of gnathosoma 40–44; palpal length 14–15; cheliceral length 48–52; *cha* 3–4; *elcp* 3–4; *h* 14–17, *h*–*h* 9; *sup* 7–8; *a* 5–6; *cm* 3–4; *ω* 6–7.

Dorsum. (Figure 1B, Figure 2C, Figure 3D,J–L, Figure 7C–F, Figure 8A–C, Figure 9A–F and Figure 10A–D). Idiosoma convex and ovoid in outline, convexity of idiosoma greater than that of male, anterior hysterosoma folds over propodosoma in specimen slide mounting; entirely sclerotized and punctate except anterior part of propodosoma and a narrow part below sejugal furrow (Figure 1B, Figure 2C, Figure 7E and Figure 9A–D); Grandjean’s organ well-developed, multi-branched with longer branches than those of male, two-parted anterior punctate supracoxal sclerites next to Grandjean’s organ; setae *scx* present small and conical, on posterior punctate supracoxal sclerites, anterior and posterior punctate supracoxal sclerites different in shape and size compared to those of male (Figure 2C, Figure 3D, Figure 7C,D and Figure 12C,D); visible remnants of missing setae *si* and *c*_1_ as rounded cupuli-form stigmata (Figure 2C and Figure 7E,F); a pair of slit-shaped cupulae *ia* visible dorsolaterally on hysterosoma, posterior to setae *c*_2_ (Figure 2C, Figure 8A, Figure 9E and Figure 10A); opisthonothal glands and their openings, *gla*, well-developed, placed near posterolateral body margin, between bases of setae *d*_2_ and *e*_2_, slightly bigger than those of male (Figure 2C, Figure 3J,K, Figure 8C and Figure 9E); a pair of unidentified pori placed posterior to seta *e*_2_ near body margin (Figure 2C, Figure 8C and Figure 9E); spermatheca and its ducts clearly visible under the tegument in mediolateral to posterior dorsal hysterosoma, spermatheca located near seta *e*_1_, inseminatory canal, twisted near copulatory opening, sclerotized parts of efferent spermaduct darker in color and trifurcate, copulatory opening ventral, located on posterior margin of opisthosoma (Figure 2C and Figure 8B,E); setae *h*_1_ observed posterior to opisthosoma; setae *h*_2_ placed posterior to setae *h*_1_, on posterior margin of opisthosoma; posterior margin of opisthosoma rounded (Figure 2C, Figure 8C and Figure 9A,B,F); all dorsal idiosomal setae simple filiform; measurements: idiosoma length 243–272, idiosoma width 150–169; propodosoma length 100–110; *vi* 12–15, *vi*–*vi* 15–16; *se* 21–26, *se*–*se* 81–82; *scx* 2–3, *scx*–*scx* 104–105; *d*_1_ 24–25, *d*_1_–*d*_1_ 42–43; *c*_2_ 19–21, *c*_2_–*c*_2_ 144–145; *d*_2_ 18–20, *d*_2_–*d*_2_ 124–125; *e*_1_ 27–30, *e*_1_–*e*_1_ 57–58; *e*_2_ 20–23, *e*_2_–*e*_2_ 110–111; *h*_1_ 24–26, *h*_1_–*h*_1_ 43–44; *h*_2_ 22–25, *h*_2_–*h*_2_ 28–29; *gla*–*gla* 112–114; *ia* length 6–7, inseminatory canal length 90–95.

Venter. (Figure 1B, Figure 2D, Figure 3G, Figure 7B, Figure 8D,E, Figure 9F, Figure 11D–F and Figure 12F). Ventral idiosoma almost flat, not entirely punctate and sclerotized in median part and opisthosoma; all coxal apodemes well-developed with fused coxal fields covered by extensive punctate shield as those of male (Figure 1B, Figure 2D, Figure 7B and Figure 9F); setae *c*_p_ placed near slit-shaped cupulae *ia* (Figure 2D, Figure 9F and Figure 10A); cupulae *im* placed in ventrolateral, slightly posterior to setae *c*_p_; cupulae *ip* positioned ventrolateral at opisthosoma margin, between legs III and IV; oviporus shaped as inverted Y, located between coxae IV; genital papillae well-developed, diameter of each genital papilla 8, anterior pair separated as well as posterior pair, by equal distance, 12; setae *g* present; placed mediolateral to genital capsule in middle-distance *4a*–*4b;* pseudanal setae absent (Figure 2D, Figure 3G, Figure 8D, Figure 11E and Figure 12F); setae *h*_3_ placed near posterior margin of opisthosoma; anal slit long, with punctate covering, extends from posterior of genital area, with a little distance, to anterior of copulatory opening at posterior margin of opisthosoma; cupulae *ih* placed anterior to setae *h*_3_, with a vertical suture on each; posterior margin of opisthosoma rounded (Figure 2D, Figure 8E, Figure 9F and Figure 11F); all ventral idiosomal setae simple filiform; measurements: *1a* 10–13, *1a*–*1a* 29–30; *3a* 12–13, *3a*–*3a* 59–60; *4a* 11–12, *4a*–*4a* 28–29; *4b* 7–9, *4b*–*4b* 11–12; *c*_p_ 17–20, *c*_p_–*c*_p_ 142–143; *g* 4–6, *g*–*g* 15–16; *h*_3_ 22–25, *h*_3_–*h*_3_ 31–32; *im*–*im* 135–137; *ip*–*ip* 104–105; *ih*–*ih* 39–40; anal slit length 51–54.

**Figure 6:**
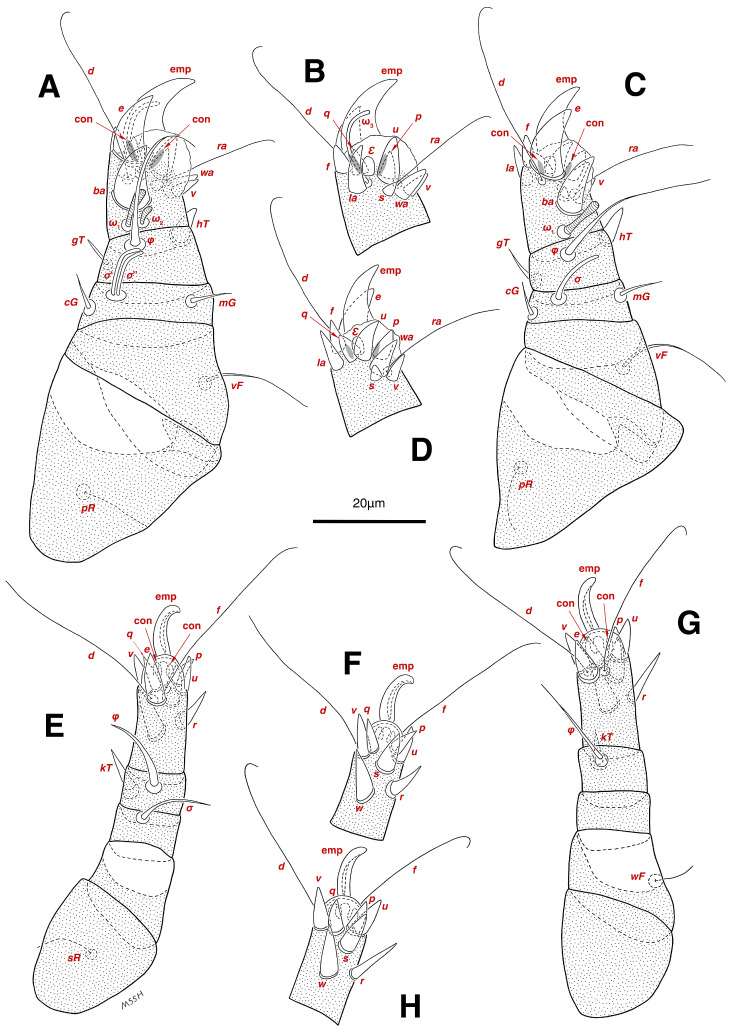
*Stereoglyphus iranensis* sp. nov., legs of female. (**A**) Leg I, dorsal view, (**B**) tarsus I, ventral view; (**C**) leg II, dorsal view, (**D**) tarsus II, ventral view; (**E**) leg III, dorsal view, (**F**) tarsus III, ventral view; (**G**) leg IV, dorsal view, (**H**) tarsus IV, ventral view. Abbreviations: emp—empodial claw, con—condylophore.

**Figure 7:**
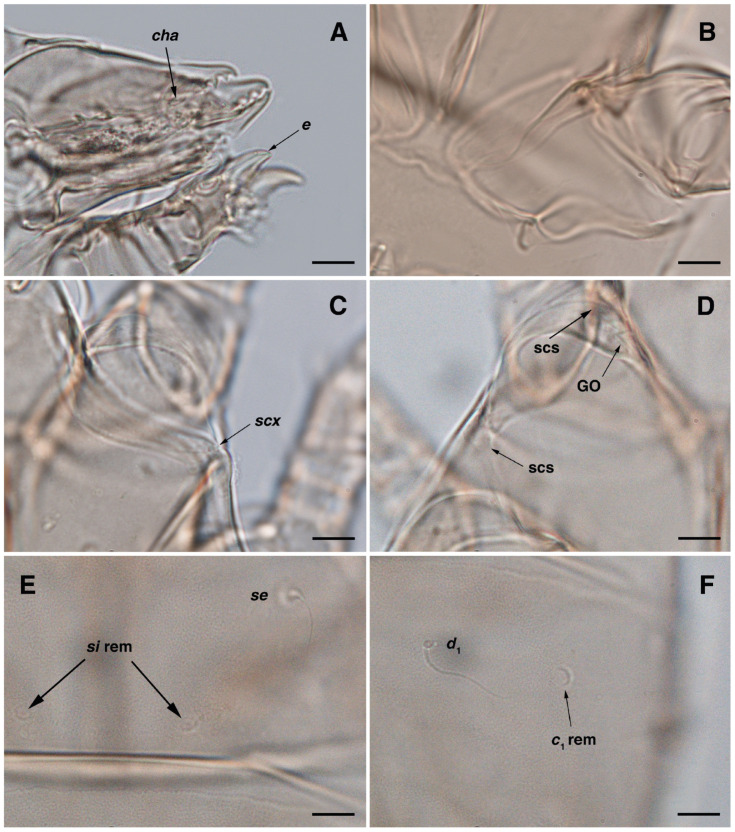
*Stereoglyphus iranensis* sp. nov., optical micrographs of female. (**A**) Chelicerae and leg I; (**B**) coxal apodeme I; (**C**) seta *scx*; (**D**) Grandjean’s organ and supracoxal sclerites; (**E**) seta *se* and remnants of setae *si*; (**F**) seta *d*_1_ and remnant of seta *c*_1_. Abbreviations: GO—Grandjean’s organ, scs—supracoxal sclerite, rem—setal remnant. Scale bar: 10 μm.

Legs. (Figure 6A–H, Figure 7A and Figure 12A–F and Table 2). Legs I–II clearly stouter and thicker than legs III–IV; pulvilli vestigial; small condylophores visible in all pretarsi; all legs punctate except parts of paraxial part of trochanters of legs I–II; empodial claws of tarsi I–II stout hooked, setae *d*, *ra* filiform, setae *e*, *ba* stout conical nearly hooked, fumuli *Ԑ* with blunt tip placed subapical, two solenidia, longer *ω*_1_ and shorter *ω*_2_ positioned basal, curved solenidia *ω*_3_ visible subapical; genual solenidia *σ*′, *σ*″ equal in length; empodial claws of tarsi III–IV long hooked, setae *f*, *d* of tarsi III–IV filiform; chaetotaxy of legs I–IV: trochanters 1-1-1-0, femora 1-1-0-1, genua 2(2*σ*)-2(1*σ*)-(1*σ*)-0, tibiae 2(1*φ*)-2(1*φ*)-1(1*φ*)-1(1*φ*), tarsi 12(3*ω*,1*Ԑ*)-12(1*ω*,1*Ԑ*)-10-10; measurements (including pulvilli, excluding empodial claws): leg I 81–86, leg II 75–80, leg III 65–70, leg IV 70–75; trochanter I 32–37, *pR* 10–12, femur I 10–14, *vF* 19–21, genu I 6–8, *σ*′ 9–10, *σ*″ 9–10, *mG* 6–7, *cG* 6–7, tibia I 8–10, *φ* 27–30, *hT* 6–7, *gT* 5–7, tarsus I 16–19, *ω*_1_ 7–8, *ω*_2_ 4–5, *ω*_3_ 14–16, *Ԑ* 3–4, *ba* 12–15, *wa* 6–7, *la* 9–10, *v* 5–6, *d* 33–36, *ra* 21–22, *e* 13–14, *q* 5–6, *p* 5–6, *u* 7–8, *f* 5–7, *s* 2–3, condylophore 4–5, empodial claw 17–19; trochanter II 34–38, *pR* 10–12, femur II 9–11, *vF* 19–21, genu II 5–7, *σ* 6–7, *mG* 7–8, *cG* 6–7, tibia II 7–9, *φ* 28–31, *hT* 6–7, *gT* 6–7, tarsus II 16–20, *ω*_1_ 7–8, *Ԑ* 3–4, *ba* 10–11, *wa* 6–7, *la* 8–9, *v* 5–6, *d* 33–35, *ra* 22–25, *e* 12–13, *q* 4–5, *p* 4–5, *f* 5–6, *s* 2–3, condylophore 3–4, empodial claw 16–19; trochanter III 22–25, *sR* 8–11, femur III 10–12, genu III 6–7, *σ* 11–13, tibia III 6–8, *φ* 14–16, *kT* 7–9, tarsus III 19–22, *w* 8–10, *r* 7–9, *d* 33–35, *f* 32–35, *e* 7–9, *p* 6–7, *q* 6–7, *u* 7–8, *v* 7–8, *s* 7–8, condylophore 2–3, empodial claw 11–13; trochanter IV 21–23, femur IV 10–13, *wF* 7–9, genu IV 7–8, tibia IV 7–8, *φ* 15–17, *kT* 6–7, tarsus IV 21–24, *w* 8–9, *r* 9–10, *d* 34–36, *f* 32–34, *e* 7–8, *p* 5–6, *q* 5–6, *u* 6–7, *v* 6–7, *s* 6–7, condylophore 2–3, empodial claw 11–12.

**Figure 8:**
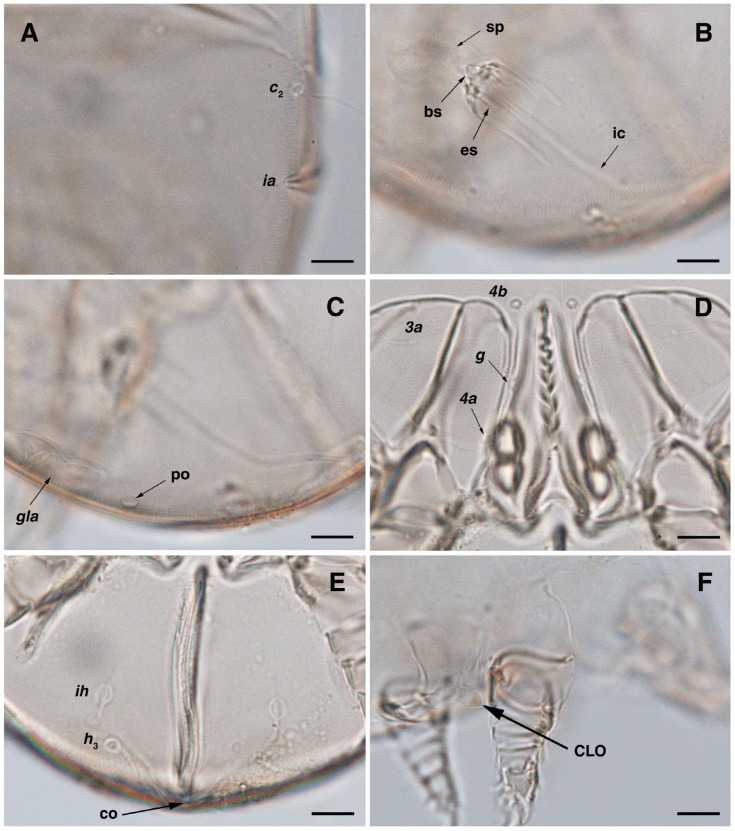
*Stereoglyphus iranensis* sp. nov., optical micrographs of female and larva. (**A**) Seta *c*_2_ and slit-shaped cupule *ia*, female; (**B**) bursa copulatrix; (**C**) opisthonothal gland opening, *gla*, and unidentified porus, female; (**D**) genital area, female; (**E**) opisthosoma, female; (**F**) Claparède organ, larva. Abbreviations: GO—Grandjean’s organ, scs—supracoxal sclerite, rem—setal remnant, sp—spermatheca, bs—base of spermatheca, ic—inseminatory canal, es—efferent spermaduct, co—copulatory opening, po—porus, CLO—Claparède organ. Scale bar: 10 μm.

**Figure 9:**
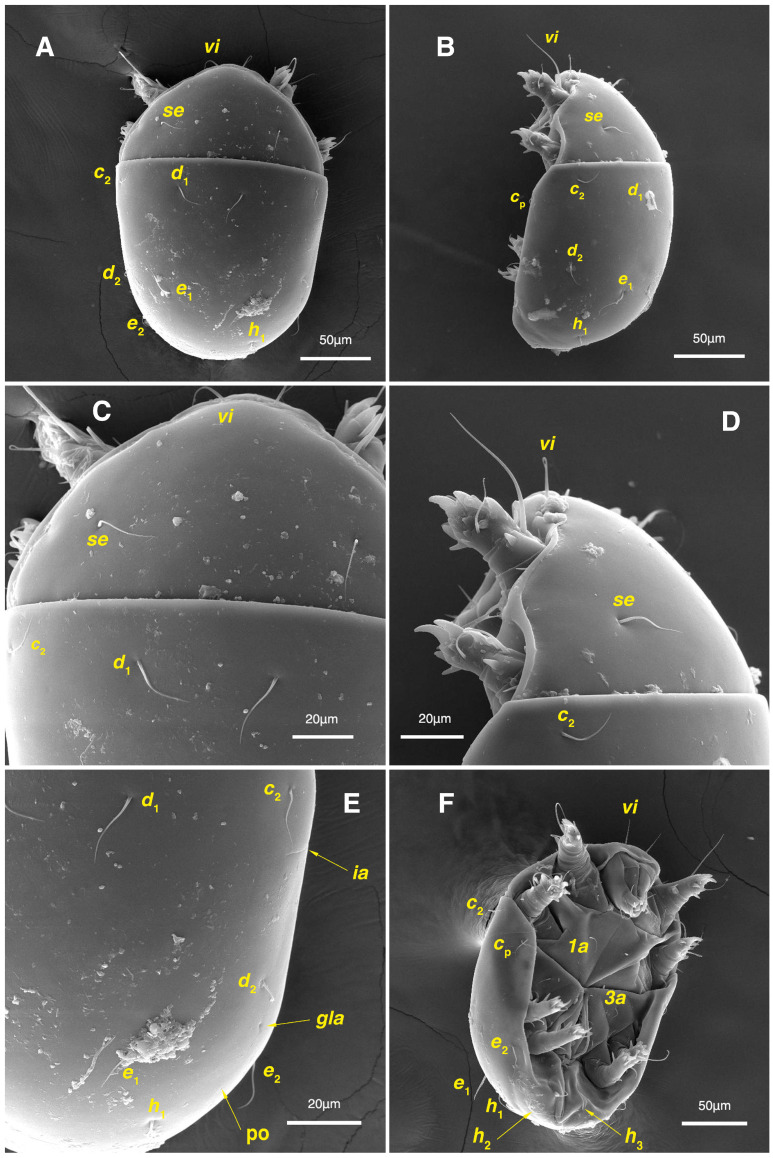
*Stereoglyphus iranensis* sp. nov., SEM micrographs of females. (**A**) Dorsal view, (**B**) lateral view; (**C**) propodosomal setae in dorsal view; (**D**) propodosomal setae in lateral view; (**E**) hysterosomal setae; (**F**) ventrolateral view. Abbreviations: po—porus.

**Figure 10:**
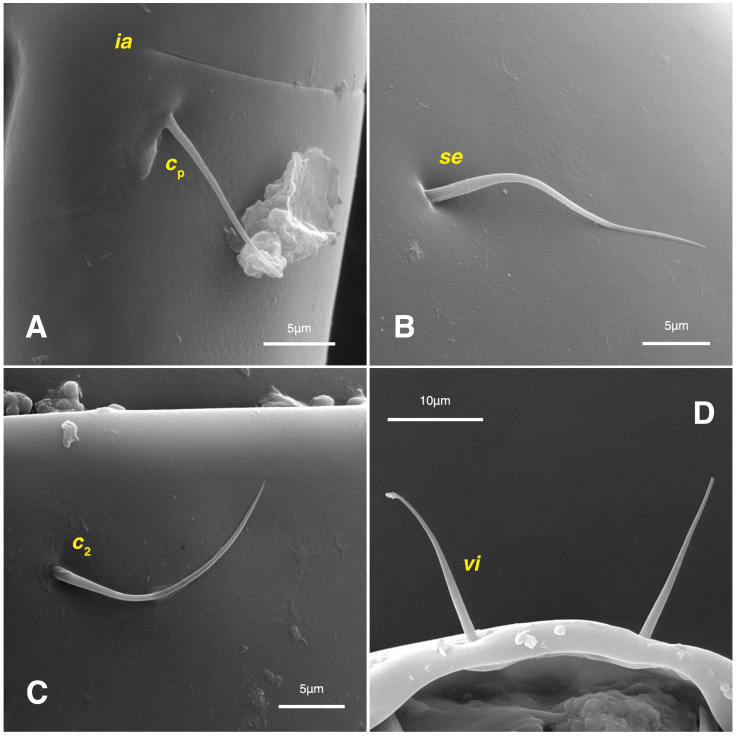
*Stereoglyphus iranensis* sp. nov., SEM micrographs of females. (**A**) Slit-shaped cupule *ia* and seta *c*_p_; (**B**) seta *se*; (**C**) seta *c*_2_; (**D**) setae *vi*.

**Figure 11:**
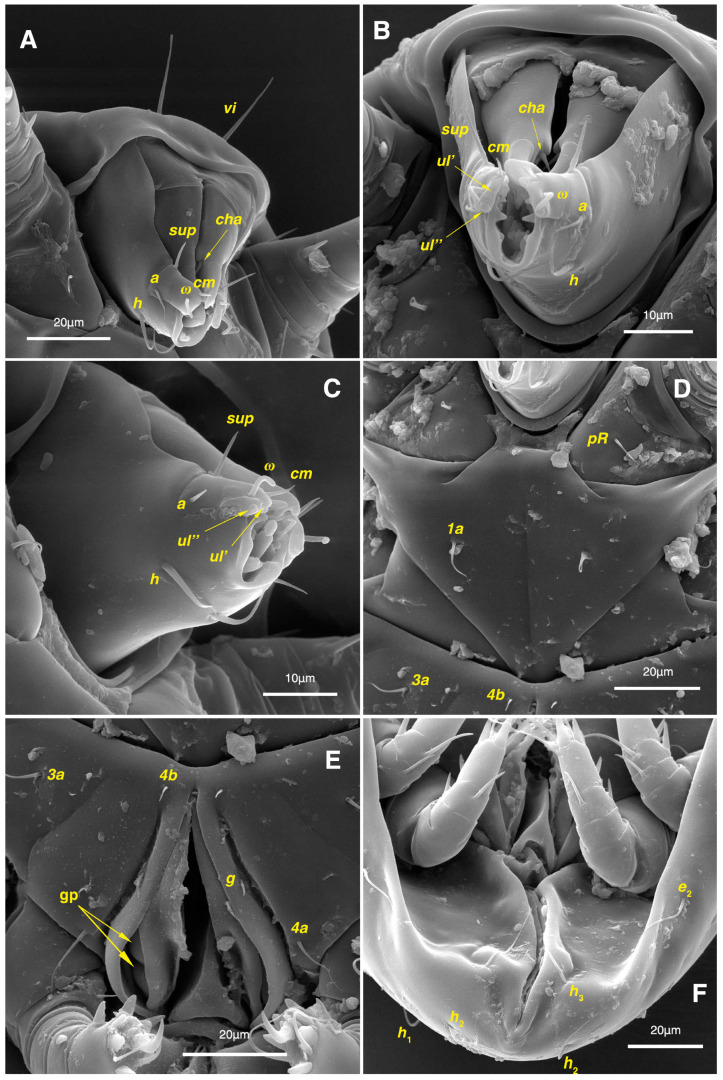
*Stereoglyphus iranensis* sp. nov., SEM micrographs of females. (**A**) Gnathosoma, dorsolateral view, (**B**) frontal view, (**C**) ventrolateral view; (**D**) coxosternal setae; (**E**) genital area; (**F**) ventral opistosoma. Abbreviation: gp—genital papillae.

**Figure 12:**
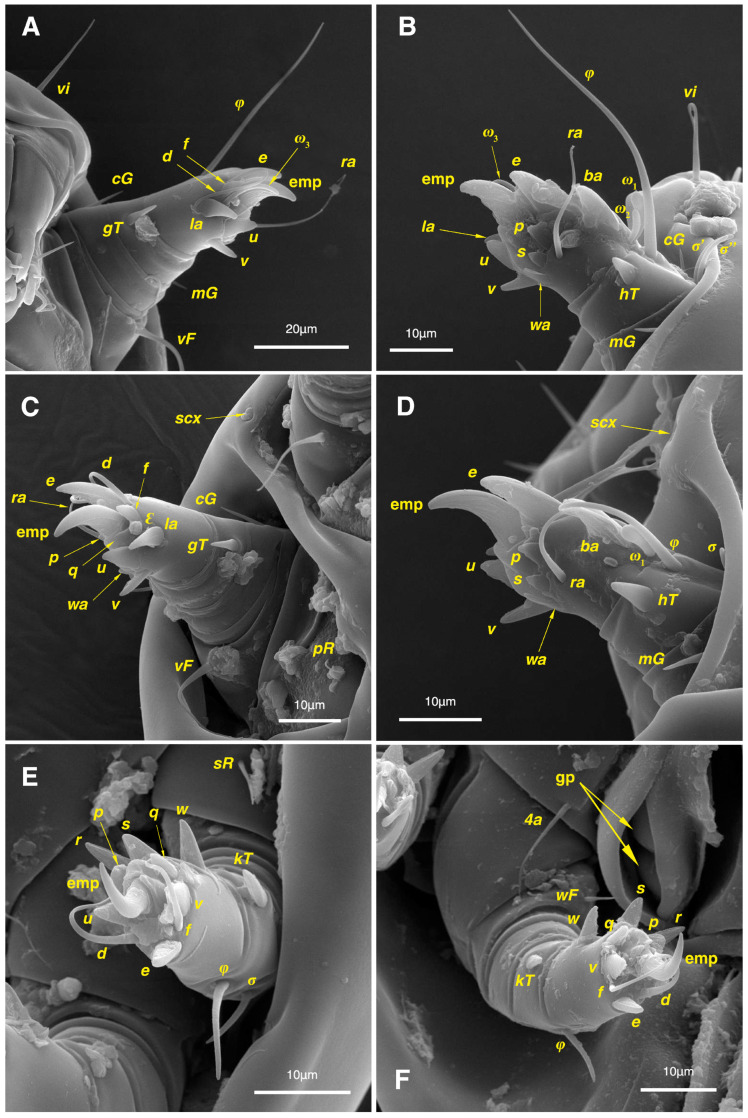
*Stereoglyphus iranensis* sp. nov., SEM micrographs of females. (**A**) and (**B**) leg I; (**C**) and (**D**) leg II; (**E**) leg III; (**F**) leg IV. Abbreviations: emp—empodial claw, gp—genital papillae.

Tritonymph (*n* = 6).

**Figure 13:**
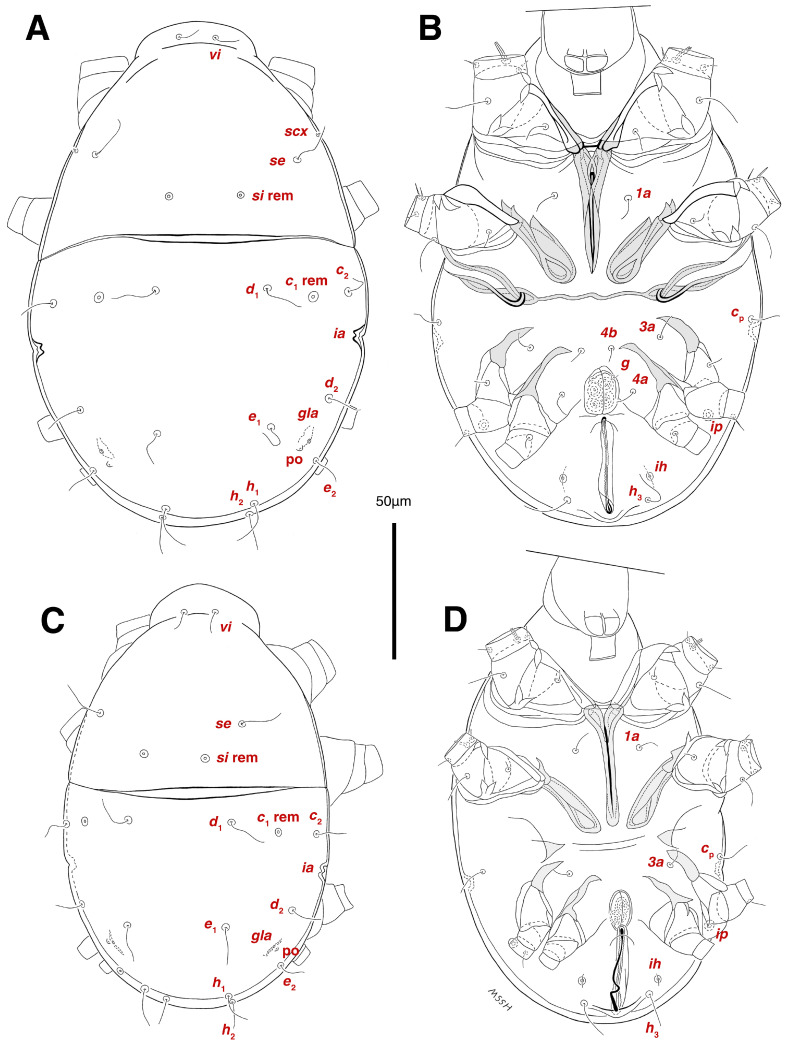
*Stereoglyphus iranensis* sp. nov., tritonymph: (**A**) dorsal view, (**B**) ventral view; protonymph, (**C**) dorsal view, (**D**) ventral view. Abbreviations: rem—setal remnant, po—porus. Segments and setae of the legs are not shown in full.

Dorsum. (Figure 1C and Figure 13A). Idiosoma convex and ovoid-shaped, convexity of idiosoma less than adult form, nearly sclerotized, not punctate; Grandjean’s organs absent; anterior and posterior punctate supracoxal sclerites not developed; very small conical setae *scx* present; remnants of missing setae *si* and *c*_1_ visible as rounded cupuli-form stigmata; a pair of slit-shaped cupule *ia* with distinct margins placed on hysterosoma, posterior to setae *c*_2_; poorly visible opisthonothal glands present with their openings, *gla*, near posterolateral body margin, between bases of setae *d*_2_ and *e*_2_; a pair of small unidentified pori located posterior to *gla*; setae *h*_1_ and *h*_2_ placed on posterior margin of opisthosoma; all dorsal idiosomal setae simple filiform; measurements: idiosoma length 166–190, idiosoma width 106–113; propodosoma length 77–81; *vi* 9–11, *vi*–*vi* 12–13; *se* 17–19, *se*–*se* 73–74; *scx* 1–2, *scx*–*scx* 88–89; *d*_1_ 14–16, *d*_1_–*d*_1_ 40–41; *c*_2_ 10–12, *c*_2_–*c*_2_ 108–109; *d*_2_ 10–13, *d*_2_–*d*_2_ 90–91; *e*_1_ 13–14, *e*_1_–*e*_1_ 41–42; *e*_2_ 11–12, *e*_2_–*e*_2_ 81–82; *h*_1_ 16–17, *h*_1_–*h*_1_ 31–32; *h*_2_ 14–15, *h*_2_–*h*_2_ 31–32; *gla*–*gla* 76–77; *ia* length 4.

Venter. (Figure 1C and Figure 13B). Ventral idiosoma flat, not punctate; coxal fields not fused; setae *c*_p_ positioned near slit-shaped cupulae *ia*; cupulae *im* absent; cupulae *ip* and *ih* present; two pairs of genital papillae developed, adjoining to each other, in a capsule; very small genital setae, *g*, present anterolateral to genital capsule in middle-distance *4a*–*4b*; anal slit extends from posterior of genital capsule, with a little distance, to posterior margin of opisthosoma; pseudanal setae absent; setae *h*_3_ placed near posterior margin of opisthosoma; all ventral idiosomal setae simple filiform; measurements: *1a* 9–10, *1a*–*1a* 26–27; *3a* 7–8, *3a*–*3a* 47–48; *4a* 6–8, *4a*–*4a* 25–26; *4b* 4–5, *4b*–*4b* 10–11; *c*_p_ 10–12, *c*_p_–*c*_p_ 114–115; *g* 2–3, *g*–*g* 7–8; *h*_3_ 15–17, *h*_3_–*h*_3_ 29–30; *ip*–*ip* 81–83; *ih*–*ih* 30–32; anal slit length 34–36.

**Figure 14:**
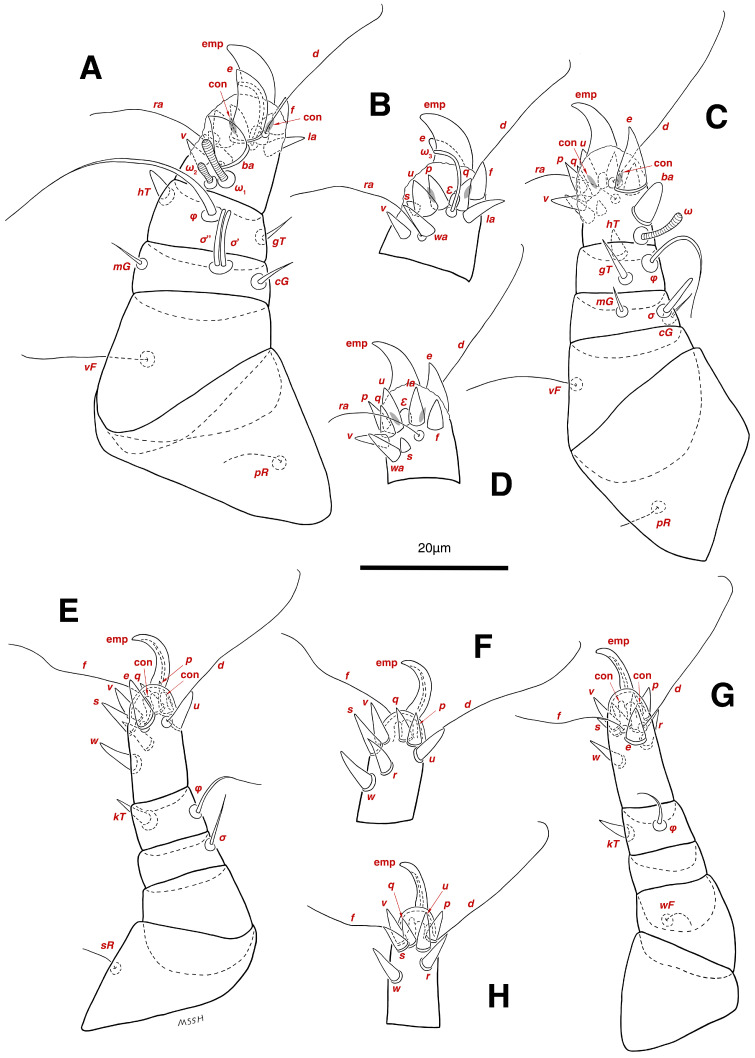
*Stereoglyphus iranensis* sp. nov., legs of tritonymph. (**A**) Leg I, dorsal view, (**B**) tarsus I, ventral view; (**C**) leg II, dorsal view, (**D**) tarsus II, ventral view; (**E**) leg III, dorsal view, (**F**) tarsus III, ventral view; (**G**) leg IV, dorsal view, (**H**) tarsus IV, ventral view. Abbreviations: emp—empodial claw, con—condylophore.

Legs. (Figure 14A–H and Table 2). Legs I–II stouter than legs III–IV; vestigial pulvilli and small condylophores visible in all pretarsi; legs not punctate; empodial claws of tarsi I–II stout hooked, setae *d*, *ra* filiform, setae *e*, *ba* stout conical nearly hooked, setae *Ԑ* with blunt tip positioned subapical, two solenidia, longer *ω*_1_ and shorter *ω*_2_ placed basal, curved solenidia *ω*_3_ subapical; genual solenidia *σ*′, *σ*″ equal in length; empodial claws of tarsi III–IV long hooked, setae *f*, *d* of tarsi III–IV filiform; chaetotaxy of legs I–IV similar to adults: trochanters 1-1-1-0, femora 1-1-0-1, genua 2(2*σ*)-2(1*σ*)-(1*σ*)-0, tibiae 2(1*φ*)-2(1*φ*)-1(1*φ*)-1(1*φ*), tarsi 12(3*ω*,1*Ԑ*)-12(1*ω*,1*Ԑ*)-10-10; measurements (including pulvilli, excluding empodial claws): leg I 62–65, leg II 56–58, leg III 52–56, leg IV 49–53; trochanter I 24–26, *pR* 5–8, femur I 10–14, *vF* 15–17, genu I 5–7, *σ*′ 8–9, *σ*″ 8–9, *mG* 5–6, *cG* 5–6, tibia I 7–9, *φ* 32–34, *hT* 4–5, *gT* 5–6, tarsus I 13–15, *ω*_1_ 5–6, *ω*_2_ 3–4, *ω*_3_ 10–12, *Ԑ* 2–3, *ba* 7–10, *wa* 4–5, *la* 5–6, *v* 4–5, *d* 22–24, *ra* 19–22, *e* 9–10, *q* 4–5, *p* 4–5, *u* 5–6, *f* 5–6, *s* 2–3, condylophore 2–3, empodial claw 13–15; trochanter II 23–25, *pR* 4–6, femur II 8–10, *vF* 14–16, genu II 4–6, *σ* 6–7, *mG* 4–5, *cG* 4–5, tibia II 7–8, *φ* 18–20, *hT* 4–5, *gT* 5–6, tarsus II 13–15, *ω*_1_ 5–6, *Ԑ* 2–3, *ba* 6–8, *wa* 5–6, *la* 5–6, *v* 4–5, *d* 24–28, *ra* 13–16, *e* 9–11, *q* 4–5, *p* 4–5, *f* 3–5, *s* 2–3, condylophore 2–3, empodial claw 12–14; trochanter III 18–20, *sR* 5–7, femur III 8–10, genu III 5–7, *σ* 6–8, tibia III 6–8, *φ* 11–13, *kT* 4–6, tarsus III 12–15, *w* 5–6, *r* 4–5, *d* 28–30, *f* 23–25, *e* 5–7, *p* 4–5, *q* 4–5, *u* 5–6, *v* 5–6, *s* 5–6, condylophore 2–3, empodial claw 9–11; trochanter IV 14–16, femur IV 8–9, *wF* 4–5, genu IV 4–5, tibia IV 5–6, *φ* 6–8, *kT* 4–5, tarsus IV 14–17, *w* 5–6, *r* 5–6, *d* 24–26, *f* 15–17, *e* 4–5, *p* 4–5, *q* 4–5, *u* 5–6, *v* 5–6, *s* 4–5, condylophore 2–3, empodial claw 9–10.

Protonymph (*n* = 3).

Dorsum. (Figure 1D and Figure 13C). Idiosoma convex and ovoid-shaped, not sclerotized nor punctate; Grandjean’s organs and setae *scx* not developed; anterior and posterior punctate supracoxal sclerites not developed; remnants of missing setae *si* and *c*_1_ visible as rounded cupuli-form stigmata; cupulae *ia* without distinct margins set on hysterosoma, posterior to setae *c*_2_; opisthonothal glands poorly visible with their openings, *gla*, near posterolateral body margin; a pair of small unidentified pori poorly visible almost at the level of *gla*, near body margin; setae *h*_1_ and *h*_2_ placed on posterior margin of opisthosoma; all dorsal idiosomal setae simple filiform; measurements: idiosoma length 153–159, idiosoma width 96–98; propodosoma length 73–75; *vi* 7–9, *vi*–*vi* 10–11; *se* 15–17, *se*–*se* 51–52; *d*_1_ 13–15, *d*_1_–*d*_1_ 37–38; *c*_2_ 9–11, *c*_2_–*c*_2_ 91–92; *d*_2_ 10–11, *d*_2_–*d*_2_ 77–78; *e*_1_ 13–14, *e*_1_–*e*_1_ 35–36; *e*_2_ 9–10, *e*_2_–*e*_2_ 58–59; *h*_1_ 12–13, *h*_1_–*h*_1_ 30–31; *h*_2_ 9–10, *h*_2_–*h*_2_ 23–24; *gla*–*gla* 58–59; *ia* length 3.

Venter. (Figure 1D and Figure 13D). Ventral idiosoma flat, not punctate; coxal fields not fused; setae *c*_p_ placed near cupulae *ia*; cupulae *im* absent; cupulae *ip* and *ih* present; a pair of small genital papillae developed, adjoining to each other, in a capsule; setae *g*, *4a*, *4b* and pseudanal setae absent; anal slit extends from posterior margin of genital capsule to posterior margin of opisthosoma; setae *h*_3_ located near posterior margin of opisthosoma; all ventral idiosomal setae simple filiform; measurements: *1a* 7–8, *1a*–*1a* 22–23; *3a* 6–7, *3a*–*3a* 36–37; *c*_p_ 10–12, *c*_p_–*c*_p_ 87–88; *h*_3_ 11–13, *h*_3_–*h*_3_ 23–24; *ip*–*ip* 71–72; *ih*–*ih* 27–28; anal slit length 31–33.

**Figure 15:**
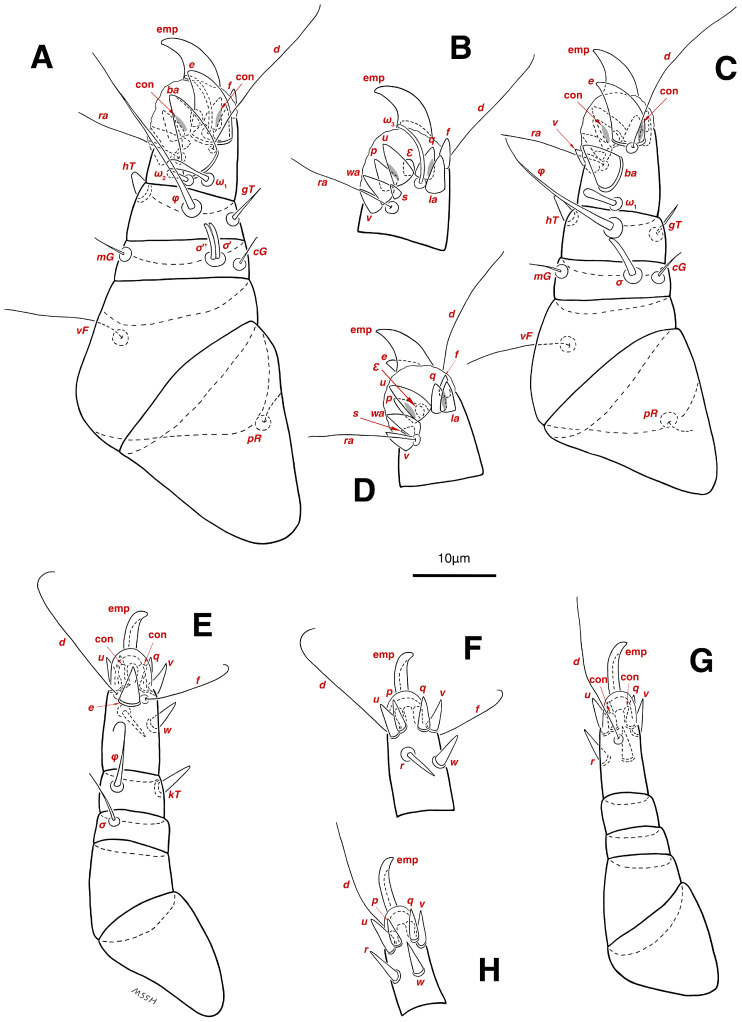
*Stereoglyphus iranensis* sp. nov., legs of protonymph. (**A**) Leg I, dorsal view, (**B**) tarsus I, ventral view; (**C**) leg II, dorsal view, (**D**) tarsus II, ventral view; (**E**) leg III, dorsal view, (**F**) tarsus III, ventral view; (**G**) leg IV, dorsal view, (**H**) tarsus IV, ventral view. Abbreviations: emp—empodial claw, con—condylophore.

Legs. (Figure 15A–H and Table 2). Legs I–II stouter than legs III–IV; vestigial pulvilli and small condylophores present in all pretarsi; legs not punctate; empodial claws of tarsi I–II stout hooked, setae *d*, *ra* filiform, setae *e* stout conical nearly hooked, short setae *Ԑ* with blunt tip positioned subapical, two solenidia, longer *ω*_1_ and shorter *ω*_2_ placed basal, curved solenidia *ω*_3_ subapical; genual solenidia *σ*′, *σ*″ equal in length; empodial claws of tarsi III–IV hooked, setae *f*, *d* tarsi III and setae *d* tarsi IV filiform; setae *s* of tarsi III, *sR* of trochanter III, *e*, *f*, *s* of tarsi IV, *kT*, *φ* of tibia IV, *wF* of femur IV, all absent; chaetotaxy of legs I–IV: trochanters 1-1-0-0, femora 1-1-0-0, genua 2(2*σ*)-2(1*σ*)-(1*σ*)-0, tibiae 2(1*φ*)-2(1*φ*)-1(1*φ*)-0, tarsi 12(3*ω*,1*Ԑ*)-12(1*ω*,1*Ԑ*)-9-7; measurements (including pulvilli, excluding empodial claws): leg I 55–58, leg II 49–51, leg III 44–47, leg IV 39–42; trochanter I 22–24, *pR* 4–6, femur I 9–11, *vF* 13–15, genu I 3–5, *σ*′ 4–5, *σ*″ 4–5, *mG* 3–4, *cG* 3–4, tibia I 4–6, *φ* 20–22, *hT* 3–4, *gT* 4–5, tarsus I 11–13, *ω*_1_ 4–5, *ω*_2_ 2–3, *ω*_3_ 7–8, *Ԑ* 2–3, *ba* 7–10, *wa* 4–5, *la* 4–5, *v* 4–5, *d* 19–22, *ra* 13–15, *e* 9–10, *q* 3–4, *p* 3–4, *u* 4–5, *f* 4–5, *s* 1–2, condylophore 1–2, empodial claw 11–13; trochanter II 20–22, *pR* 3–5, femur II 7–9, *vF* 11–13, genu II 3–5, *σ* 5–6, *mG* 3–4, *cG* 3–4, tibia II 6–7, *φ* 16–18, *hT* 3–4, *gT* 4–5, tarsus II 12–14, *ω*_1_ 4–5, *Ԑ* 1–2, *ba* 6–8, *wa* 5–7, *la* 4–5, *v* 3–4, *d* 20–22, *ra* 11–13, *e* 7–9, *q* 3–4, *p* 3–4, *f* 2–3, *s* 1–2, condylophore 1–2, empodial claw 10–12; trochanter III 14–16, femur III 6–8, genu III 4–6, *σ* 5–6, tibia III 5–7, *φ* 10–12, *kT* 4–5, tarsus III 13–14, *w* 3–4, *r* 3–4, *d* 19–22, *f* 10–12, *e* 3–5, *p* 3–4, *q* 3–4, *u* 4–5, *v* 4–5, condylophore 1–2, empodial claw 6–8; trochanter IV 10–12, femur IV 7–8, genu IV 3–4, tibia IV 4–5, tarsus IV 9–12, *w* 4–5, *r* 4–5, *d* 18–21, *p* 3–4, *q* 3–4, *u* 4–5, *v* 4–5, condylophore 1–2, empodial claw 8–9.

Larva (*n* = 1).

**Figure 16:**
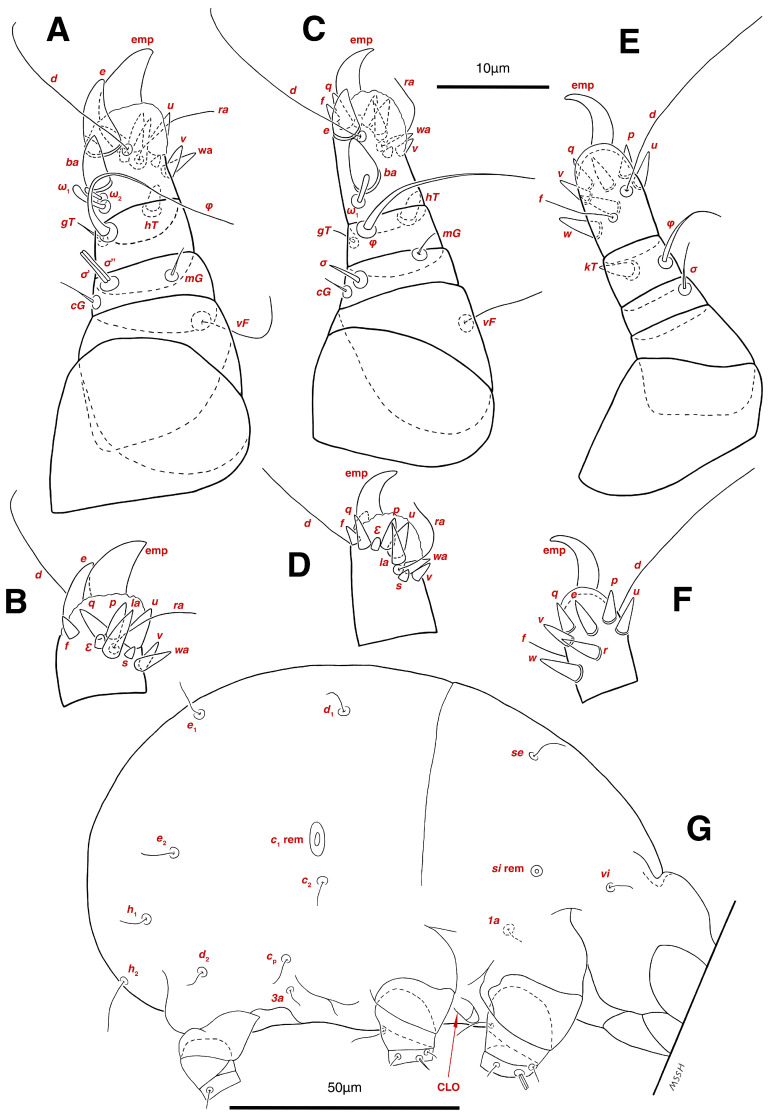
*Stereoglyphus iranensis* sp. nov., larva. (**A**) Leg I, dorsal view, (**B**) tarsus I, ventral view; (**C**) leg II, dorsal view, (**D**) tarsus II, ventral view; (**E**) leg III, dorsal view, (**F**) tarsus III, ventral view; (**G**) lateral view. Abbreviations: emp—empodial claw, rem—setal remnant, CLO—Claparède organ.

Latus. (Figure 1E, Figure 8F and Figure 16G and Table 2). Idiosoma convex and ovoid-shaped in dorsum, flat in venter; not sclerotized nor punctate; sejugal furrow thin line; Grandjean’s organ, supracoxal sclerites, setae *scx*, cupulae *ia*, *im*, *ip*, *ih*, setae *4a*, *4b*, *g*, *h*_3_, opisthonotal glands and their openings, *gla*, all absent; dorsal and ventral idiosomal setae simple filiform; measurements: idiosoma length 125, idiosoma width 78; propodosoma length 62; Claparède organ length 6; *vi* 5, *se* 8, *d*_1_ 6, *c*_2_ 5, *d*_2_ 5, *e*_1_ 8, *e*_2_ 7, *h*_1_ 6, *h*_2_ 12, *1a* 4, *3a* 4, *c*_p_ 6.

Legs. (Figure 16A–F and Table 2). Legs I–II stouter than leg III; vestigial pulvilli present in all pretarsi; condylophores not observed; legs not punctate; empodial claws tarsi I–II stout hooked, tarsi III long hooked; setae *d*, *ra* of tarsi I–II and setae *d* of tarsus III filiform; setae *e* stout conical on tarsi I–II, nearly hooked just on tarsi I; short setae *Ԑ* with blunt tip placed subapical of tarsi I–II; two solenidia, longer *ω*_1_ and shorter *ω*_2_ located basal tarsi I; solenidia *ω*_3_ not observed; genual solenidia *σ*′, *σ*″ equal in lenght; setae *s* of tarsi III, *pR* of trochanter I, *pR* of trochanter II, *sR* of trochanter III, all absent; chaetotaxy of legs I–III: trochanters 0-0-0, femora 1-1-0, genua 2(2*σ*)-2(1*σ*)-(1*σ*), tibiae 2(1*φ*)-2(1*φ*)-1(1*φ*), tarsi 12(2*ω*,1*Ԑ*)-12(1*ω*,1*Ԑ*)-9; measurements (including pulvilli, excluding empodial claws): leg I 39, leg II 33, leg III 37; trochanter I 17, femur I 4, *vF* 10, genu I 4, *σ*′ 4, *σ*″ 4, *mG* 3, *cG* 3, tibia I 5, *φ* 19, *hT* 2, *gT* 3, tarsus I 9, *ω*_1_ 3, *ω*_2_ 2, *Ԑ* 1, *ba* 5, *wa* 3, *la* 5, *v* 3, *d* 16, *ra* 9, *e* 7, *q* 3, *p* 3, *u* 4, *f* 2, *s* 1, empodial claw 9; trochanter II 11, femur II 4, *vF* 7, genu II 3, *σ* 3, *mG* 2, *cG* 3, tibia II 4, *φ* 17, *hT* 3, *gT* 2, tarsus II 11, *ω*_1_ 3, *Ԑ* 1, *ba* 5, *wa* 3, *la* 4, *v* 2, *d* 18, *ra* 8, *e* 4, *q* 3, *p* 3, *f* 2, *s* 1, empodial claw 7; trochanter III 14, femur III 5, genu III 3, *σ* 4, tibia III 4, *φ* 8, *kT* 3, tarsus III 9, *w* 4, *r* 3, *d* 20, *f* 6, *e* 3, *p* 3, *q* 3, *u* 3, *v* 3, empodial claw 7.

**Table 2:** Changes in leg chaetotaxy in *Stereoglyphus iranensis* sp. nov. during ontogeny.

Life stages	Legs	Trochanters	Femora	Genua	Tibiae	Tarsi
Larva	I	-	*vF*	*cG*, *mG*, *σ*′, *σ*″	*gT*, *hT*, *φ*	*ba*, *f*, *e*, *d*, *la*, *wa*, *ra*, *s*, *q*, *p*, *u*, *v*, *ω*_1_, *ω*_2_, *ε*
	II	-	*vF*	*cG*, *mG*, *σ*	*gT*, *hT*, *φ*	*ba*, *f*, *e*, *d*, *la*, *wa*, *ra*, *s*, *q*, *p*, *u*, *v*, *ω*_1_, *ε*
	III	-	-	*σ*	*kT*, *φ*	*f*, *e*, *d*, *w*, *r*, *q*, *p*, *u*, *v*
Protonymph	I	*pR*	*vF*	*cG*, *mG*, *σ*′, *σ*″	*gT*, *hT*, *φ*	*ba*, *f*, *e*, *d*, *la*, *wa*, *ra*, *s*, *q*, *p*, *u*, *v*, *ω*_1_, *ω*_2_, *ω*_3_, *ε*
	II	*pR*	*vF*	*cG*, *mG*, *σ*	*gT*, *hT*, *φ*	*ba*, *f*, *e*, *d*, *la*, *wa*, *ra*, *s*, *q*, *p*, *u*, *v*, *ω*_1_, *ε*
	III	-	-	*σ*	*kT*, *φ*	*f*, *e*, *d*, *w*, *r*, *q*, *p*, *u*, *v*
	IV	-	-	-	-	*d*, *w*, *r*, *q*, *p*, *u*, *v*
Tritonymph	I	*pR*	*vF*	*cG*, *mG*, *σ*′, *σ*″	*gT*, *hT*, *φ*	*ba*, *f*, *e*, *d*, *la*, *wa*, *ra*, *s*, *q*, *p*, *u*, *v*, *ω*_1_, *ω*_2_, *ω*_3_, *ε*
	II	*pR*	*vF*	*cG*, *mG*, *σ*	*gT*, *hT*, *φ*	*ba*, *f*, *e*, *d*, *la*, *wa*, *ra*, *s*, *q*, *p*, *u*, *v*, *ω*_1_, *ε*
	III	*sR*	-	*σ*	*kT*, *φ*	*f*, *e*, *d*, *w*, *r*, *s*, *q*, *p*, *u*, *v*
	IV	-	*wF*	-	*kT*, *φ*	*f*, *e*, *d*, *w*, *r*, *s*, *q*, *p*, *u*, *v*
Adult	I	*pR*	*vF*	*cG*, *mG*, *σ*′, *σ*″	*gT*, *hT*, *φ*	*ba*, *f*, *e*, *d*, *la*, *wa*, *ra*, *s*, *q*, *p*, *u*, *v*, *ω*_1_, *ω*_2_, *ω*_3_, *ε*
	II	*pR*	*vF*	*cG*, *mG*, *σ*	*gT*, *hT*, *φ*	*ba*, *f*, *e*, *d*, *la*, *wa*, *ra*, *s*, *q*, *p*, *u*, *v*, *ω*_1_, *ε*
	III	*sR*	-	*σ*	*kT*, *φ*	*f*, *e*, *d*, *w*, *r*, *s*, *q*, *p*, *u*, *v*
	IV	-	*wF*	-	*kT*, *φ*	*f*, *e*, *d*, *w*, *r*, *s*, *q*, *p*, *u*, *v*

**DNA barcoding.** From the 11 female paratypes of *Stereoglyphus iranensis* sp. nov. used for DNA extraction and PCR, a 661 bp COI gene was successfully sequenced for 7 specimens. All studied specimens shared the same COI haplotype (GenBank acc. nos. PX696963–696969). A BLAST search of the COI sequences in the GenBank database shows no records for the genus *Stereoglyphus*. This study represents the first DNA barcode data for this genus.

**Differential diagnosis.**

Females of *S. iranensis* sp. nov. differ from females of *S. haemisphaericus* Berlese, 1923, in shorter idiosoma length (243–270 vs. 280), idiosoma width (150–169 vs.180); in setae *h* distances, *h*_1_–*h*_1_ = *h*_2_–*h*_2_ vs. *h*_1_–*h*_1_ 44, *h*_2_–*h*_2_ 29; females of *S. iranensis* sp. nov. differ from females of *S. luciae* (Fain, 1966), in shorter idiosoma width (150–169 vs. 184–189), *vi* (12–14 vs. 15–20), *se* (21–25 vs. 30), longer inseminatory canal (90–95 vs. 60–70); females of *S. iranensis* sp. nov. differ from females of *S. subterraneus* (Fain, 1976), in shorter idiosoma length (243–270 vs. 290), idiosoma width (150–169 vs. 183), cheliceral length (48–52 vs. 83), *se*–*se* (82 vs. 120), longer *d*_1_ (24–25 vs. 10–12), *c*_2_ (19–21 vs. 15), *e*_1_ (27–30 vs. 10–12), inseminatory canal (90–95 vs. 50); male of *S. iranensis* sp. nov. differs from males of *S. longibursatus* (Fain et Mahunka, 1990), in shorter idiosoma width (124 vs. 147–160), *vi* (9 vs. 18–20), *h*_3_ (22 vs. 25), longer propodosomal length (90 vs. 80), *se* (18 vs. 12), *d*_1_ (22 vs. 12–15), *c*_2_ (12 vs. 5–8), *d*_2_ (17 vs. 5–8), *e*_1_ (19 vs. 12–15), *e*_2_ (19 vs. 15), *h*_1_ (20 vs. 12–15), *h*_2_ (18 vs. 12–15); females of *S. iranensis* sp. nov. differ from females of *S. longibursatus* (Fain et Mahunka, 1990), in shorter idiosoma width (150–169 vs. 180–202), inseminatory canal (90–95 vs. 115–120).

### 3.3. Additional Record of the Genus Stereoglyphus

New record of *S. longibursatus* (Fain et Mahunka, 1990).

**Material examined.** One female specimen, Iran, Fars Province, Darab County, Chah Kondar Village, Sahlak Cave; 28°32′51.20″ N, 55°08′33.80″ E; mixture of soil and bat guano; 4 December 2021; leg. M. Sadat-Shojaei.

**Depository.** One female, microscopic slide, ZM-CBSU.

### 3.4. Species Distribution of the Genus Stereoglyphus

**Figure 17:**
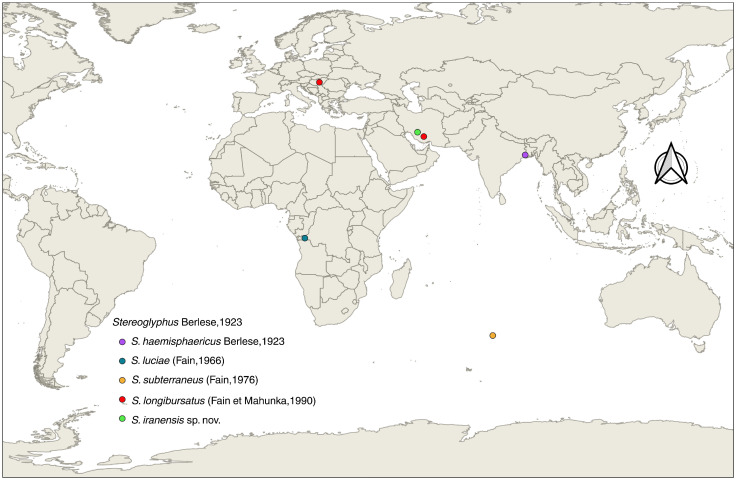
Global distribution of *Stereoglyphus* species; visualization using QGIS ver. 3.44.3-Solothurn.

Based on the geographical distribution map of known *Stereoglyphus* species (Figure 17), this study established the second Palearctic record of this genus and the first Asian record of *S. longibursatus*. Therefore, this genus and these two species are new to the astigmatid acarofauna of Iran. Hence, the species of this genus are as follows: *S. haemisphaericus* Berlese, 1923, *S. luciae* (Fain, 1966), *S. subterraneus* (Fain, 1976), *S. longibursatus* (Fain et Mahunka, 1990) and *S. iranensis* sp. nov.

### 3.5. Key to Species of the Genus Stereoglyphus

The key applies only to females, as males of *S. haemisphaericus* and *S. luciae* are unknown.

1. Idiosoma length of 280 μm or more …………………………….…….…………………………………………………………………………………………………2
– Idiosoma length less than 280 μm …………………………….…….……………………………………………………………………………………………………3
2. *h*_1_–*h*_1_ equal to *h*_2_–*h*_2_ ……...…………………….…….…………………………………………………………………………………*S. haemisphaericus* Berlese, 1923
– *h*_1_–*h*_1_ not equal to *h*_2_–*h*_2_ ……...………………………………………………………………………………………………………………*S. subterraneus* (Fain, 1976)
3. Filiform setae *scx* present; female inseminatory canal length of 115 μm or more ……………………………………*S. longibursatus* (Fain et Mahunka, 1990)
– Setae *scx* not filiform or absent; female inseminatory canal length of 100 μm or less ……………………………………………………………………………..4
4. Setae *scx* absent; posterior margin of opisthosoma conical; length of inseminatory canal in female 60–70 μm …………………………*S. luciae* (Fain, 1966)
– Conical small setae *scx* present; posterior margin of opisthosoma rounded; length of inseminatory canal in female 90–95 μm …………………………….…….………………………………………………………………………………………………………………………*S. iranensis* sp. nov.

## 4. Discussion

Troglobionts are obligate cave-dwelling animals restricted to the dark zone (hypogean) of caves [25,26,27,28]. Specimens of *Stereoglyphus iranensis* sp. nov., collected from a mixture of soil and bat guano in the hypogean zone of Doroodzan Cave, conform to this definition. The same substrate was also sampled in illuminated zones near the cave entrance, but no specimens of this species were found. As a part of the cave-dwelling Acari identification project in the Zagros Mountains, sampling was conducted in more than 20 caves, and *S. iranensis* sp. nov. was not recorded from any other locality. This species therefore represents the second troglobiont astigmatid mite species recorded from caves of the Zagros Mountains, Iran, following the description of *Ciprusenia troglobionta* Sadat-Shojaei et Haitlinger, 2023 which belongs to the family Canestriniidae [10]. In the case of *S. longibursatus*, sampled from the hypogean zone of Sahlak Cave, only a single female specimen was collected. As the type locality of this species is in Hungary, further studies are required to clarify its taxonomic and ecological status in Iran.

Two aspects of the *Stereoglyphus* morphology are particularly noteworthy: the specific form and location of the famuli *Ԑ* on the tarsi of the anterior legs, and the reduction of dorsal setae on the propodosoma (*si*) and the anterior part of the hysterosoma (*c*_1_). Of all sensory organs of the actinotrichid tarsi, we know least about the function of famuli. The famuli are placed on tarsi I, rarely on tarsi II and most often are peglike, but also umbellate, stellate, or may be hidden in a small pit [29,30,31]. Since the role of this seta is unknown, it has been referred by Grandjean as “a mysterious organ” [32]. Its structure is characterized by the presence of a terminal porus, which suggests that it may function as a contact chemoreceptor (gustatory sensilla); however, its minute size makes this interpretation unlikely [30].

The famuli were reported from numerous species of the subfamily Rhizoglyphinae and in all cases they are short, peglike and placed on tarsi I at the base or close to solenidia *ω*_1_ [33,34,35,36]. In the case of *Stereoglyphus iranensis* sp. nov., famuli are observed on tarsi I and II of all life stages, positioned subapically near the empodial claws. They also have an unusual appearance—a short and thick protuberance with a blunt tip. The unusual shape of the famulus clearly refers to almost all other tarsal setae on legs I-II which are modified into short and thick spines (except for the hair-like *d* and *ra*); even the solenidia are shortened. This setal structure, together with robust front legs resembling mole paws, is also observed in certain other rhizoglyphin genera (*Acarotalpa*, *Schwiebea*) and is presumably an adaptation to burrowing in the substrate [37]. It is likely that the reduction of the dorsal setae of the idiosoma is also an adaptation to squeezing between particles of guano lying inside caves. The reduction of idiosomal dorsal setae can often be the result of purely mechanical factors, as is commonly observed, for example, in feather mites subjected to physical pressure from the host’s rubbing feathers and aeration [38].

These morphological observations should be proven by future analyses of the microanatomy and behavior of *Stereoglyphus* and related genera.

## 5. Conclusions

Cave-dwelling acariform mites remain insufficiently studied in many Iranian caves located in the Zagros Mountains. The limited accessibility of numerous caves in this mountain range has significantly hindered comprehensive faunistic surveys, and consequently many cave-dwelling animals, particularly mites, remain unidentified. In addition to morphological and molecular identification of subterranean mites, studying various aspects of their biology is challenging because of the unique conditions of the cave ecosystem. In the present study, the fifth species of the genus *Stereoglyphus* Berlese, 1923 is described on the basis of morphological analyses supported by SEM and DNA barcode data (COI). The new species, collected from the dark zone of Doroodzan Cave, Fars Province, Iran, is hypothesized to represent a troglobitic mite adapted to life in bat guano deposits. This interpretation is supported by distinct morphological modifications, including shortened and robust anterior legs with most tarsal setae transformed into stout spines (“mole-like” legs) and a partial reduction of dorsal idiosomal setation. These findings highlight the need for further integrative studies combining taxonomy, molecular data, and ecological observations to elucidate the evolutionary pathways of cave-associated mites.

## Data Availability

The data presented in this study are available on request from the corresponding authors.
